# Oxidative Stress-Mediated Aging during the Fetal and Perinatal Periods

**DOI:** 10.1155/2014/358375

**Published:** 2014-08-18

**Authors:** Lucia Marseglia, Gabriella D'Angelo, Sara Manti, Teresa Arrigo, Ignazio Barberi, Russel J. Reiter, Eloisa Gitto

**Affiliations:** ^1^Neonatal and Pediatric Intensive Care Unit, Department of Pediatrics, University of Messina, Via Consolare Valeria 1, 98125 Messina, Italy; ^2^Unit of Pediatric Genetics and Immunology, Department of Pediatrics, University of Messina, Via Consolare Valeria 1, 98125 Messina, Italy; ^3^Department of Cellular and Structural Biology, University of Texas Health Science Center at San Antonio, San Antonio, TX 40729, USA

## Abstract

Oxidative stress is worldwide recognized as a fundamental component of the aging, a process that begins before birth. There is a critical balance between free radical generation and antioxidant defenses. Oxidative stress is caused by an imbalance between the production of free radicals and the ability of antioxidant system to detoxify them. Oxidative stress can occur early in pregnancy and continue in the postnatal period; this damage is implicated in the pathophysiology of pregnancy-related disorders, including recurrent pregnancy loss, preeclampsia and preterm premature rupture of membranes. Moreover, diseases of the neonatal period such as bronchopulmonary dysplasia, retinopathy of prematurity, necrotizing enterocolitis, and periventricular leukomalacia are related to free radical damage. The specific contribution of oxidative stress to the pathogenesis and progression of these neonatal diseases is only partially understood. This review summarizes what is known about the role of oxidative stress in pregnancy and in the pathogenesis of common disorders of the newborn, as a component of the early aging process.

## 1. Introduction

Aging is an inevitable natural phenomenon mainly characterized by increased oxidative stress (OS), elevated inflammatory responses, accelerated cellular senescence, and progressive organ dysfunction that lead to a gradual decline in the physical and mental faculties of individuals. In aging, these homeostatic imbalances significantly alter cellular responses to injury [1]. Identification of factors that regulate aging is limited by the complexity of the process and by its considerable heterogeneity among individuals. At the cellular level, the most prominent event in an aging tissue is cell senescence, a consequence of exposure to intrinsic and extrinsic aging factors, characterized by gradual accumulation of DNA damage and epigenetic changes in DNA structure that modulate correct gene expression leading to altered cell function [2].

Aging is a multifactorial process that is determined by genetic and environmental factors. The genotype determines the variation in lifespan among species or individuals. Many aging disorders, including atherosclerosis, diabetes, and hypertension, result from years of impact of a combination of environmental assaults and genetic susceptibility [3]. Although there has been a great deal of focus on the genes that determine aging, the nongenetic regulation of aging is gaining increased attention. Specifically, aging is associated with profound epigenetic changes, resulting in alterations of gene expression and disturbances in broad genome architecture and the epigenomic landscape [4]. Overall, more than 300 theories have been proposed to explain the aging process [5], but none has yet been generally accepted by gerontologists. However, the free radical theory of aging seems to be the one receiving the widest acceptance as a plausible explanation of the primary cell biological changes that are basis of the aging process [6]. Partially in contrast with the OS related theory of aging evidence has emerged in recent years where reactive oxygen species (ROS) may actually work as essential and potentially lifespan-promoting, signaling molecules which transduce signals from the mitochondrial compartment to other organelles of the cell [7, 8]. Low-level oxidative damage eventually culminates in increased stress resistance and ultimately in longevity. This reflects an adaptive response commonly defined as hormesis [9] and better named mitochondrial hormesis or mitohormesis, which refers to ROS-related stress emanating from the mitochondria [10, 11].

Regarding the free radical theory of aging, OS, which is enhanced by smoking and bad dietary habits, is recognized as a fundamental underlying component of the aging process. The changes result in hyperactivity of progrowth factors, accumulation of toxic molecular aggregates, and activation of death/survival pathways which culminate into cellular apoptosis or necrosis [1]. OS and proinflammatory processes are strongly related [12, 13]. Upon activation, many immune cells generate free radicals (FR) and, equally, overproduction of partially reduced oxygen species induces an inflammatory response.

Since OS occurs precociously before conception, it can be stated that the aging processes begin before birth [14]. OS clearly occurs early in pregnancy and continues in the postnatal period. This molecular damage contributes to the pathophysiology of many disorders during pregnancy, at childbirth, and during the neonatal period. Pregnancy, in fact, is a state associated with enhanced OS related to high metabolic turnover and elevated tissue oxygen requirements [15]. During intrauterine life, many factors such as hypoxia, inflammation, and infections can induce overproduction of FR [16]. Whenever produced, these toxic species can induce OS and tissue injury. The placenta represents an important source of oxidatively damaged lipid peroxides, because of its high polyunsaturated fatty acid content [17]. Circulating levels of peroxidation markers, such as lipid hydroperoxide and malondialdehyde, are higher in pregnant than in nonpregnant women [18] and these products are elevated in the second trimester and decrease after delivery [19].

OS occurs when the production of toxic FR exceeds the capacity of defensive mechanisms to neutralize them. There is normally a critical balance between FR generation and antioxidant defenses. FR reactions lead to the oxidation of lipids, proteins, and polysaccharides and to DNA fragmentation, base modifications, and strand breaks; as a result, radicals have a wide range of biologically destructive effects [20, 21]. The placenta is the exchange organ between the mother and the developing fetus. Adequate functioning of this organ is clearly essential for a proper progress of gestation and the development of a healthy offspring as final outcome. As with every developing organ, the placenta has an adaptive capacity to cope with variations in maternofetal conditions, with short and long-lasting responses resulting in potential morphostructural and functional changes [22].

Placental function and fetal development are dependent on oxygen availability and limiting ROS generation. In fact, overproduction of ROS causes altered placental remodeling and abnormal fetal development and growth. The mechanisms involved not only impact fetal life, but also may mediate long-lasting effects in the newborn [23, 24]. The programming effects on the fetoplacental unit due to intrauterine stress and the potential mechanisms are currently under intensive research. The proposed main modulating mechanisms are epigenetic, such as DNA methylation and histone deacetylation [25].

Newborns, especially if preterm, are particularly vulnerable to OS because they exhibit accelerated production of FR and limited antioxidant protection, which increases the susceptibility of rapidly growing tissues to damage [26, 27]. These “FR-related diseases” of neonates promote cellular, tissue, and organ damage. Oxygen therapy, often required in the neonatal period, exaggerates OS since oxygen is a proven source of FR. In 1988, Saugstad coined the phrase “oxygen radical disease in neonatology” to highlight the crucial role of OS in a wide range of neonatal disorders [28].

This review summarizes what is known about the role of OS in healthy and complicated pregnancies and in the pathogenesis of common diseases of the newborn, as a component of the early aging process.

## 2. Oxidative Stress and Pregnancy-Related Disorders

Pregnancy is a state of metabolic challenge to be met by the mother and the developing fetus and, even under normal conditions, it is associated with an elevated levels of OS compared to that what occurs during the nonpregnant state [18, 19]. Due to its high metabolic rate and level of elevated mitochondrial activity, the placenta is a key source of this OS [29, 30]. Intrauterine OS during pregnancy is a physiologic response to fetoplacental energy demands [31]. Placental tissues contain low concentrations and activities of antioxidant enzymes during the first trimester, and so, trophoblastic cells are particularly susceptible to oxygen-mediated damage [32]. During the first trimester the oxygen tension within the intervillous space is about 20 mmHg [33], and the placental tissues display low activities of the principal antioxidant enzymes including catalase, glutathione peroxidase, and Cu/Zn and Mn superoxide dismutase [32]. Concentrations are particularly low in the syncytiotrophoblast, and so this tissue is especially vulnerable to OS. Thus, when the oxygen tension raises threefold in the intervillous space with the onset of maternal arterial flow at the start of the second trimester, a burst of OS is observed in the placenta. This oxidative injury could alter placental remodeling and function, affecting the subsequent course of gestation [34]. The generation of FR is an intrinsic result of aerobic energetics, but the process is well balanced in healthy pregnancy by enzymatic and nonenzymatic antioxidant redox systems [35]. The ability of placental antioxidant defenses to reduce the effects of highly reactive and potentially damaging FR is critical for healthy placental function and optimal growth and development of the fetus. Occasionally, however, the developing fetus may be exposed to high levels of OS due to overproduction of FR and a reduced antioxidant defense capability [33].

Abnormal placentation in the first trimester leads to OS and the resultant endothelial dysfunction plays a key role in the emergence of complications of pregnancy, such as recurrent abortions and preeclampsia [32]. OS, in fact, has emerged as a likely promoter of several pregnancy-related disorders including preeclampsia, fetal growth restriction (FGR), recurrent pregnancy loss (RPL), preterm birth (PTB), and preterm prelabor rupture of the membranes (pPROM) [36].

Preeclampsia is a serious multisystem syndrome and a major cause of maternal, fetal, and neonatal morbidity and mortality that complicates approximately 5% of all pregnancies and 10% of all first pregnancies. This condition is associated with elevated oxidatively damage molecules in the maternal circulation. Placental OS resulting from the ischemia-reperfusion injury is involved in the pathogenesis of preeclampsia [37]. A significant rise in lipid peroxidation levels in the placenta of preeclamptic women has been demonstrated [38]. Excess iron levels and a decreased unsaturated iron binding capacity are factors associated with OS and contribute in the pathogenesis of preeclampsia/eclampsia. Negi et al. [39] demonstrated a significant elevation in the levels of 8-hydroxydeoxyguanosine, protein carbonyls, nitrite and iron along with reduced levels of catalase, vitamins A, E, and C, and total antioxidant status in the umbilical cord blood of preeclamptic and eclamptic pregnancies. Also, the production of the antioxidant melatonin is significantly depressed in the placenta of preeclamptic women [40]. These parameters might be influential variables for the risk of FR damage in infants born to preeclamptic/eclamptic pregnancies [39].

It has been demonstrated that OS is associated with FGR; in fact, this condition is often complicated by intrauterine hypoxia and impaired blood flow to the fetus [41]. A chronic restriction in uterine blood flow elicits placental and fetal responses in the form of growth adaptation to hypoxia. Intrauterine hypoxia may induce FR generation and, therefore, fetal OS [42, 43].

OS also plays a critical role in the etiopathogenesis of RPL. Safronova et al. [44] found an elevated production of toxic oxygen species in the granulocytes of patients with a history of RPL versus control subjects with normal reproductive function. Increased lipid peroxidation and reduced antioxidant levels in women with a history of abortions were reported by Şimşek et al. [45], supporting the concept that OS plays a central role in the etiopathogenesis of RPL. Inflammatory immune responses also are a key factor in reproductive failures, such as in multiple implantation failures, early pregnancy loss, and RPL, because cellular immune responses, especially those mediated by natural killer and T cells, are often dysregulated in these conditions [46].

OS is a postulated etiology of spontaneous PTB and pPROM, although the precise mechanistic role of FR in these complications is unclear. Animal studies have shown that decidual senescence can lead to PTB by activating p53, a proapoptotic factor, and inflammatory cytokines [47, 48]. Recent biomolecular and histologic data on pPROM and PTB suggest that increased ROS and oxidative damage to lipids and DNA in fetoplacental cells play an important pathophysiological role in these disorders [49, 50].

Argüelles et al. [51] measured OS markers including protein carbonyl groups, lipid peroxides, and total antioxidant capacity and found a good correlation between the oxidative state of the normal mother and the neonate, with a high level of maternal OS corresponding to an even higher OS in newborn umbilical cord blood.

Recently, the use of antioxidant therapies has attracted much attention. Vitamin C, an antioxidant which reacts with superoxide and lipid hydroperoxide radicals, has been studied, but data from large randomized controlled trials do not support the routine supplementation of pregnant women with high doses of the vitamins C or E [52, 53]. The reasons are unclear. First, it is possible that although OS plays a major role in the pathophysiology of preeclampsia, it is not the only causal pathway of the disorder. On the other hand, OS could be relevant to the pathogenesis of preeclampsia in only a subgroup of women, with no appreciable benefit of vitamins C and E for the entire population [54]. In addition, vitamin C and E, although considered antioxidants, can act as conditional prooxidants increasing OS paradoxically. Prooxidative reactions can be greatly enhanced in the presence of transition metal ions [55]. In the presence of redox-cycling oxidizing agents such as Fe, Cu, or Cr, antioxidant protection might take on a prooxidative form because of Fenton-like chemistry [56].

Encouraging results have been reported, however, after the administration of the antioxidant melatonin [57, 58], in both pregnant women and newborns [59]. Melatonin has a great capacity to scavenge radicals and reduce oxidative damage in the placenta [60] by increasing antioxidative enzymes and decreasing lipid peroxidation [61]. It neutralizes many more toxic reactants than vitamins C or E [58, 62] and acts as an indirect antioxidant by stimulating antioxidative enzymes [63]. Moreover, melatonin synthesis occurs in the placenta and the villous trophoblasts contain the classic transmembrane receptors for the indole, MT1, and MT2 [64]. To document this, villous cytotrophoblasts were isolated from human term placentas after vaginal delivery, demonstrating that the villous cytotrophoblasts and the syncytiotrophoblasts from the human placenta also contain the two enzymes, serotonin N-acetyltransferase and N-acetylserotonin methyltransferase, which metabolize serotonin to melatonin [64]. Locally generated melatonin functions in the protection of the placenta from OS employing both receptor-dependent and receptor-independent processes. Furthermore, this hormone reduces the loss of villous cytotrophoblasts by preventing apoptosis of these cells [65]. Also, when human villous trophoblast cells from term placentas of uncomplicated pregnancies were isolated and treated with melatonin, the placental trophoblast was protected against hypoxia-reperfusion-induced OS and apoptosis [66, 67]. Owing to its strong antioxidant capacity and its ability to cross the placenta and blood-brain barrier [68, 69], melatonin is of particular interest as a candidate for antenatal neuroprotectant [70]. In the human and animal models, melatonin treatment before and during transient severe fetal asphyxia reduces oxidative damage to the fetal brain [71], reduces lipid peroxidation [72] and cell death, and stabilizes the blood-brain barrier [73, 74].

The relationship between the oxidative status of the mother and the newborn at the moment of birth has been investigated. During the transition from fetal to neonatal life at birth, the fetus is transferred from an intrauterine hypoxic environment with an oxygen tension (PO_2_) of 20–25 mmHg to an extrauterine normoxic environment with a PO_2_ of 100 mmHg. This increase induces an elevated production of ROS as well as reactive nitrogen species (RNS) [75]. Oxygen, vital to survival, is highly damaging to fetal tissues which are known to be poorly equipped to neutralise its toxic derivatives. Although, the resuscitation of newborn infants was traditionally performed with pure oxygen [76, 77], it is currently recognized that OS is elevated when resuscitation is performed with 100% oxygen [78]. The American Heart Association guidelines focused on the optimal management of oxygen during neonatal resuscitation, emphasizing that either insufficient or excessive oxygenation can be harmful to the newborn infant [79].

## 3. Oxidative Stress and Neonatal Diseases

Increased levels of OS and reduced antioxidant capacities may contribute to the pathogenesis of disorders in the perinatal period and excessive formation of ROS may be related to morbidity. Newborns, especially when born prematurely, are very susceptible to FR-mediated oxidative damage for several reasons: (a) infants at birth are naturally exposed to the hyperoxic challenge due to the transition from the hypoxic intrauterine environment to extrauterine life, and this difference is even more significant for newborns who require supplemental oxygen during resuscitation in the delivery room; (b) infants are more susceptible to infection, especially if born prematurely; (c) they have reduced antioxidant defense processes; (d) they possess high levels of free iron that enhances the Fenton reaction causing the production of the highly toxic hydroxyl radical [80, 81].

The contribution of OS to the pathogenesis and progression of neonatal diseases is only partially understood, and the statement “oxygen radical disease in neonatology” has been proposed to underline the key role of OS in a wide range of neonatal morbidities [28] including hypoxic ischemic encephalopathy (HIE) [20], intraventricular hemorrhage (IVH) [82], periventricular leukomalacia [83], bronchopulmonary dysplasia (BPD), chronic lung disease (CLD) [84], retinopathy of prematurity (ROP) [85], and necrotizing enterocolitis (NEC) [86]. Subsequently, it became clear that FR influences the ductus arteriosus and pulmonary circulation [87, 88]. If the concept of “oxygen radical diseases of neonatology” is valid, it might mean that the abovementioned conditions are not different disease entities but are simply different organ manifestations of the same complex processes of OS and metabolism.

The fetal and neonatal brain are particularly vulnerable to the effects of oxygen and nitrogen-based FR. OS is implicated in the pathogenesis of many neurological diseases such as IVH [89], HIE [90], and epilepsy [91]. The high concentration of unsaturated fatty acids in the neonatal brain predisposes to the propagation of OS. The propensity of the preterm brain to respond to the dangerous effects of OS relates not only to the deficient antioxidant defenses, but also to several prooxidant characteristics; in fact, developing neural tissues have a high metabolic rate supported almost exclusively by oxidative metabolism, which is an important source of FR. The degree of damage depends on the region of the brain that is affected, the severity of the injury, and the stage of development. The morbidity and mortality of infants are strongly affected by their inability to maintain physiologic homeostasis and to counteract the effects of toxic oxygen derivatives. When an inflammatory response is initiated during a perinatal brain injury, it is associated with the induction of a variety of cytokines, including TNF-*α*, IL-1, and IL-6 [92, 93]. A similar cytokine response has been found to be involved in several neurodegenerative diseases that typically affect humans in old age [94–96].

Elevated OS and/or reduced endogenous antioxidant defenses may also play a role in the pathogenesis of a number of inflammatory pulmonary diseases including respiratory distress syndrome (RDS) in the newborn [97, 98]. Oxygen, which is obviously vital to survival, can be highly damaging to tissues such as neonatal lungs, which are known to be poorly equipped to neutralize oxygen's toxic derivatives. ROS and RNS induce ultrastructural changes in the cytoplasm of pulmonary capillary endothelial cells and cause focal hypertrophy thereby altering metabolic activity. ROS also have been implicated in the molecular damage seen in the bronchoalveolar lavage (BAL) fluid of patients with RDS [99, 100]. In fact, hydrogen peroxide is detected in the expired air of RDS patients, and myeloperoxidase and oxidized-1-antitrypsin have been found in BAL fluid. CLD or BDP is a general term for long-term respiratory problems in premature babies. The underling mechanisms of BPD are not clearly defined, although OS in ventilated infants with immature lungs is crucial component of the injury process that leads to CLD [101]. Barotrauma, volutrauma, and oxygen toxicity during mechanical ventilation are assumed to be important factors in the pathogenesis of CLD as they cause pulmonary damage, resulting in a release of multiple proinflammatory cytokines, including IL-6, IL-8, TNF-*α*, and the production of extracellular matrix components and growth factors [102, 103]. Balinotti et al. [104] also found that surface area for diffusion in the lung increased during the first 2 years of life and is proportional to alveolar volume. In BPD patients, an impaired alveolar growth, decreased pulmonary diffusion capacity, and susceptibility to develop chronic obstructive pulmonary disease phenotype with aging have been reported. Premature senescence might be a common physiological response to pulmonary OS in children and adults [105].

Yet another neonatal disease in which the OS plays a critical role is ROP, a proliferative condition of the retinal vasculature in premature infants that may cause severe visual loss; it is more common in premature infants exposed to high concentrations of oxygen as it causes generation of FR. Hypoxia as well is also known to be involved in the genesis and progression of ROP [106, 107]. In animal studies, intermittent hypoxia induced a more severe form of ROP than equally distributed hypoxemic episodes [108]. Di Fiore et al. [106] demonstrated that an increase in the prevalence of hypoxemic episodes during the first 8 weeks of life in preterm newborns is correlated to severe ROP requiring laser therapy. The pathologic blood vessel proliferation causing blindness in proliferative retinopathy of premature infants is also the main cause of visual impairment in diabetic retinopathy, one of the most common complications of diabetes in working age adults [109].

NEC is a gastrointestinal tract disorder of premature neonates that results in inflammation and bacterial invasion of the bowel wall. Many etiologic factors including immaturity, hypoxia/ischemia, hyperosmolar feedings, bacterial colonization, and OS have been identified [110]. Perrone et al. [111] measured nonprotein bound iron and total hydroperoxides in cord blood and showed that the determination of OS biomarkers was useful in identifying babies at high risk for NEC.

## 4. Conclusions 

FR, normally produced in living organisms, are highly reactive molecules containing one or more unpaired electrons. If they are produced in excess, they are important mediators of cell and tissue injury [112], causing or accelerating senescence processes. Physiological pregnancies and even more so complicated pregnancies represent a condition of increased exposure to FR. Furthermore, we emphasize the strong correlation between the oxidative status of the normal mother and the neonate, showing that a high maternal OS level corresponds to even higher OS in the newborn, with accelerated cellular senescence. Infants have limited protective mechanisms against OS, which contributes to maternal and neonatal disorders [37, 80, 113]. The critical perinatal susceptibility to OS indicates that prophylactic use of antioxidants could help to prevent or at least reduce OS-related diseases in pregnancy and in newborns. Recently, the use of the antioxidant melatonin has been proposed to protect fetus and newborn against perinatal OS [59]; this molecule also has a very high safety profile [114] further supporting its use.

In conclusion, the scientific evidence concerning OS in development of pregnancy complication and several diseases of the newborn confirm that aging process starts at the time of conception.
